# B Cells and Autoantibodies in AIRE Deficiency

**DOI:** 10.3390/biomedicines9091274

**Published:** 2021-09-21

**Authors:** Anette S. B. Wolff, Sarah Braun, Eystein S. Husebye, Bergithe E. Oftedal

**Affiliations:** 1 Department of Medicine, Haukeland University Hospital, 5021 Bergen, Norway; anette.boe@uib.no (A.S.B.W.); sarah_braun95@gmx.de (S.B.); Eystein.husebye@uib.no (E.S.H.); 2 Department of Clinical Science, University of Bergen, 5021 Bergen, Norway; 3 KG Jebsen Center for Autoimmune Disorders, University of Bergen, 5021 Bergen, Norway; 4 Institute of Pharmacy and Molecular Biotechnology, Ruprecht-Karls University, 69120 Heidelberg, Germany

**Keywords:** autoimmune polyendocrine syndrome type 1 (APS-1), autoantibodies, B-cells, B-cell dependent therapy, mouse models of Aire deficiency

## Abstract

Autoimmune polyendocrine syndrome type 1 (APS-1) is a rare but severe monogenetic autoimmune endocrine disease caused by failure of the Autoimmune Regulator (AIRE). AIRE regulates the negative selection of T cells in the thymus, and the main pathogenic mechanisms are believed to be T cell-mediated, but little is known about the role of B cells. Here, we give an overview of the role of B cells in thymic and peripheral tolerance in APS-1 patients and different AIRE-deficient mouse models. We also look closely into which autoantibodies have been described for this disorder, and their implications. Based on what is known about B cell therapy in other autoimmune disorders, we outline the potential of B cell therapies in APS-1 and highlight the unresolved research questions to be answered.

## 1. Introduction

Autoimmune polyendocrine syndrome type 1 (APS-1), also known as autoimmune polyendocrinopathy–candidiasis–ectodermal dystrophy (APECED), is a monogenetic autoimmune disease with an estimated prevalence of 1:100,000 caused by mutations in the *autoimmune regulator* (*AIRE*) gene [1,2,3,4,5]. Patients usually develop autoimmune manifestations in multiple organs leading to functional impairment, especially of various endocrine glands. APS-1 is diagnosed by the presence of two out of three major clinical components of hypoparathyroidism, chronic mucocutaneous candidiasis (CMC), and primary adrenocortical insufficiency or Addison’s disease. Besides these, multiple other manifestations occur, like primary ovarian insufficiency (POI), autoimmune thyroid disease, type 1 diabetes (T1D), autoimmune gastritis, keratitis, vitiligo, alopecia and enamel hypoplasia are common [1,6]. Alternatively, finding *AIRE* mutations and specific autoantibodies can be used diagnostically and enable early diagnosis before the main components develop [2,7].

The disease-causing mutations in *AIRE* are typically inherited in an autosomal recessive manner [8,9], although several heterozygous missense mutations have also been found with a dominant-negative inheritance pattern [10,11]. Overall, more than 130 mutations in *AIRE* have been identified (Human Gene Mutation Database, www.hgmd.cf.ac.uk, accessed on the 10 of May 2021), many of which cluster in key domains of the AIRE protein [2,8,12,13,14,15,16]. AIRE is mainly expressed in a subset of thymic medullary epithelial cells (mTECs), regulating the expression of 20% of the 20,000 unique tissue-restricted antigens (TRAs) to be presented to the developing T cells during negative selection [17,18,19,20,21]. This transcription factor contributes to the development of thymic Foxp3+ CD4+ regulatory T cells (Tregs) and is crucial for their ability to re-circulate back to the thymus [22,23,24]. In addition, AIRE is necessary for the generation of Tregs ex-thymus [25]. Indeed, APS-1 patients have decreased numbers of Tregs with a modified TCR repertoire compared to healthy individuals, reflecting the abnormal selection of T cells in the thymus [26,27].

Although the pathogenic mechanisms in APS-1 are T cell-mediated, the B cells are important antigen-presenting cells (APCs) that rely on T cell activation for the production of antibodies. They are found in the thymus and are also reported to have some AIRE expression themselves [28,29,30]. We will here look closer into what is known about the B cells in APS-1 patients and AIRE-deficient mouse models, summarize the status quo and the outstanding research questions, and highlight the therapeutic strategies involving B cells in APS-1.

## 2. B Cell’s Contribution to APS-1 and Aire Deficiency

Even though the loss of negative selection of T cells in the thymus also affects B cells, both in the thymus and beyond [31,32,33], the role of B cells and their autoantibodies in the pathogenesis of APS-1, as well as their therapeutic potential, is still incompletely known. However, exemplified by B cell depleting therapies, the role of B cells in APS-1 is likely to be more pronounced than previously assumed. One study revealed that treatment with Rituximab, a monoclonal antibody targeting CD20 and leading to B cell depletion, caused a significant reduction of inflammation, infiltration, and tissue destruction in Aire knockout mice [34]. In a study by Popler and co-workers, an APS-1 patient with severe pulmonary disease was treated with Rituximab after the failure of several other immunomodulating therapy approaches, whereupon lung function improved [35]. One could speculate if this effect is tissue-dependent and rely on the observed B cell expansion in the airway mucosal tissue upon antigen exposure [36]. If this also indicates that B cells and T cells contribute differentially to the different disease components remains to be answered. Several human studies have demonstrated recovery of APS-1-related disease components through B cell depletion by Rituximab. This includes studies on patients with isolated autoimmune Addison’s disease where depletion of B cells alone or in combination with depot tetracosactide led to an increase of cortisol and aldosterone levels in a subgroup of participants [37,38].

The autoantibodies produced by B cells are unlikely to be directly pathogenic; rather, they serve a mediating role in the context of an infectious milieu. This is underpinned by experiments showing that sera from AIRE-deficient mice cannot transfer autoimmunity [39]. The autoantibodies are however excellent diagnostic markers, as will be discussed later.

## 3. Thymic B Cells and Their Interaction with Developing T Cells

The B cell antigen receptor (BCR) has two roles in B cell activation; first as a binder of antigen that transmits signals directly to the cell’s interior, and secondly by internalizing the antigen for degradation and subsequent presentation on the B cell surface as peptides bound to MHC class II molecules MHC-II. Antigen-specific helper T cells recognize the peptide:MHC-II complex and produce cytokines that cause the B cell to proliferate and its progeny to differentiate into antibody-secreting cells. Somatic hypermutation and switching to certain immunoglobulin isotypes depend on the interaction of antigen-stimulated B cells with helper T cells and other cells in the peripheral lymphoid organs [40]. There is a bidirectional relationship between B and T cells and a requirement for sequential input from both cell types to generate a successful overall outcome. It is suggested that the beneficial effect of B cell depletion in autoimmune settings could be explained by harnessing unfavorable T cell activation [41].

In the thymus, tissue-resident B cells are found at a frequency of 0.1–1% [42,43,44,45,46], likely reflecting the homing of peripheral B cells and their potential as APCs. In murine model systems, the thymic B cells have been found to be potent APCs able to present TRAs induced by Aire expression. Although dendritic cells (DCs) and mTECs are the major acting APCs, recent data argue that B cells have a specific role in this process, as mice lacking either B cells, DCs or had depressed MHC-II expression have an equal CD4+ single positive compartment, i.e., they are equally pivotal for intra-thymic antigen presentation [29,47]. B cells also modulate the production and maintenance of thymic Tregs [48,49]. Of interest, B cells within the thymus can expand and undergo class switching, thereby directly influence CD4 T cell tolerance. In human thymi, B cells are found in the medulla and throughout the cortex and include all developmental stages with an overweight of mature naïve B cells [28]. They have been shown to express AIRE, and the expression level declines with increasing age [28,50].

## 4. Loss of Aire and the Consequences for B Cells Ex-Thymus

B cells have their own tolerance mechanisms which are mainly achieved within the bone marrow by clonal deletion of autoreactive cells and receptor editing to become functional receptors and to maximize recognition towards their cognate antigen [51]. The immature B cells migrate to secondary lymphoid organs where they further develop to mature B cells expressing IgM and IgD. B cells that bind their antigens and get proper T cell help in concert with CD40-CD40L binding will proliferate and differentiate into memory B cells or plasma cells that secrete antibodies [52]. Antibodies produced by plasma cells have three major roles in the host defense: neutralization of pathogens, opsonization for phagocytosis, and complement activation. In peripheral tolerance, anergy is induced in the remaining self-reactive B cells by lack of antigen interaction and hence co-stimulation by T cells [17,53].

Central B cell tolerance is described as functional in APS-1 patients, reflecting that it is established independently of T cells and their AIRE-dependent selection [26]. The tolerance mechanisms in the periphery are however impaired, resulting in a peripheral accumulation of autoreactive mature naïve B cells. Hence, adequate T cell tolerance is crucial for obtaining optimal peripheral B cell selection. An Aire-deficient mouse model with impairment of the hematologic development of the monocyte linage developed marginal zone B cell (MZB) lymphoma, indicating an exaggerated activation of B cells in AIRE-deficient mice which increased with age [54]. The development of MZB lymphoma has further been linked to increased levels of B cell-activating factor of the TNF family (BAFF) produced by DCs in the periphery upon IFN-α stimulation, highlighting that AIRE regulates T cell-independent B cell responses through BAFF [55]. BAFF was also increased in APS-1 patients in two other reports [26,56] suggesting B cell impairment as this factor is required for peripheral B cell survival and homeostasis and for regulating the expression of the B cell essential receptor CD21.

Number-wise, there are conflicting reports regarding the peripheral B cell subsets found in APS-1 patients. The frequency of CD19+ cells in the blood is reported to be similar to those in controls [57] or slightly fewer [31,56,58]. Sng et al. showed that the B cell subpopulations were in the normal reference range in the AIRE-deficient state, except for the CD19+CD27−CD21−/lo compartment which was expanded [26]. Another study claimed age-dependent lower levels of switched-memory (CD27+ IgM−) B cells, but an increase in CD27+ IgM+ memory B cells [31]. These findings indicate first that the B cell phenotype is significantly altered in APS-1 patients and secondly, that the innate immune system, represented by the circulating MZB cells and the IgM positive B cells, contributes to the disease progression. Specific information about the autoreactive B cells would aid to move the field forward.

## 5. Autoantibodies in APS-1

A hallmark of APS-1 is multiple high-titer circulating autoantibodies, some of which are excellent diagnostic markers of both the disease itself and different disease components (Table 1). The autoantibodies in APS-1 can be classified into two groups: organ-specific autoantibodies as well as systemic autoantibodies against certain cytokines of the immune system itself.

The most common organ-specific autoantigens include 21-hydroxylase (21OH) correlating to Addison’s disease, NOD-like receptor pyrin domain containing 5 (NALP5) associated to both hypoparathyroidism and POI, and side-chain cleavage enzyme (SSC) in females, also linked to ovarian insufficiency [1]. Other autoantibodies are frequently seen although the correlation to specific manifestations is less clear: tyrosine hydroxylase, aromatic L-amino acid decarboxylase (AADC) and tryptophan hydroxylase (TPH) [12]. These autoantibodies are useful diagnostic markers in addition to the clinical diagnosis, ensuring an autoimmune component to the disease. Of note, these autoantibodies are also seen in patients with isolated disease components, e.g., the detection of 21OH in patients with isolated Addison’s disease.

The most frequently found autoantibodies target cytokines and have neutralizing properties in vitro. Ninety-five percent of APS-1 patients display autoantibodies against IFN-ω and IFN-α [59], while interleukin-17 (IL-17) family cytokines, especially IL-22, are targeted in around 90 % of all patients [60], making these excellent screening tools to identify APS-1 patients. Overall, the most common autoantigens in APS-1 are IFN-ω, IL-22 and 21OH [2,59], which are regularly used as diagnostic tools preceding AIRE sequencing. An overview of APS-1 autoantigens as well as the corresponding clinical manifestations are given in Table 1.

**Table 1:** Overview of the most important autoantigens and associated clinical manifestations in APS-1.

Clinical Manifestation	Autoantigen	Ref
Chronic mucocutaneous candidiasis	IL-22, IL-17	[60,61]
Hypoparathyroidism	NALP5, CaSR	[62,63]
Addison’s disease	21OH	[64,65]
Ovarian failure	SSC, 17OH, NALP5	[66,67]
Testicular failure	TSGA1, TGM4, PDILT, MAGEB2, SSC	[68,69,70]
Type 1 diabetes	Insulin, IA2	[71]
Autoimmune hepatitis	CYP1A2, AADC, TPH	[72,73,74]
Intestinal malabsorption	TPH	[72,74]
Vitiligo	SOX-9, SOX-10, AADC	[75,76]
Alopecia	TH	[77]
Pulmonary disease	KCNRG, BPIFB1	[78,79]
Non-organ specific	IFN-ω, IFN-α2, IL-22, IL-17	[60,61,80]

17OH, 17 hydroxylase; 21OH, 21 hydroxylase; AADC, aromatic L-amino acid decarboxylase; BPIFB1, bactericidal/permeability-increasing fold-containing B1; CYP1A2, Cytochrome P450 1A2; GAD65, glutamic acid decarboxylase 65; IA2, tyrosine phosphatase like protein; IL, interleukin; IFN, interferon; KCNRG, potassium channel regulating protein; MAGEB2, melanoma-associated antigen B2; NALP5, NOD-like receptor pyrin domain containing 5; PDILT, protein disulphide isomerase-like, testis expressed; SOX, SRY-related HMG-box; SSC, side-chain cleavage enzyme; TGM4, transglutaminase 4; TH, tyrosine hydroxylase; TPH, tryptophan hydroxylase.

The autoantibodies seen in APS-1 are a result of the lack of peripheral tolerance as the autoantibodies against IL-17A, IL-17F, and IL-22 amongst others were shown to be present in the bone marrow pool in both APS-1 patients and controls. However, while these markers for autoimmunity were still present in the peripheral B cell pool of APS-1 patients, they were removed in healthy subjects, indicating this essential immune controls step is deficient in APS-1 patients [26]. The mechanisms leading to the high titers of autoantibodies presenting early in life are still unclear. Autoantibodies against IFN-ω can be found as early as 6 months of age [81], resembling the early presence of autoantibodies in Immune dysregulation, polyendocrinopathy, enteropathy, X-linked (IPEX) syndrome, that are often detectable from birth [82]. Additionally, autoantibodies targeting IFN-α have been reported in a subgroup of IPEX patients [83]. The loss of Tregs and consequently, peripheral T cell tolerance mechanisms in IPEX patients leads to the accumulation of peripheral B cells and autoantibodies. This underlines the effect loss of T cell tolerance, either central or peripheral, implies on B cell function leading to autoimmune disease and immunodeficiency. The IFN-α autoantibodies have also recently been described in critically ill COVID-19 patients, in comparison to those with uncomplicated disease courses. This study concluded that a B cell autoimmune phenocopy of inborn errors of type I IFN immunity explained life-threatening COVID-19 pneumonia in 2.6% of females and 12.5% of males [84]. Looking to APS-1 patients having undergone COVID-19 infection, the picture is less clear, as both mild and severe COVID-19 infections have been reported [85,86,87,88].

A few antigens have been reported to be targeted by autoantibodies as well as autoreactive T cells. Mapping the autoreactive T cell epitopes is a cumbersome work, in particular in humans where the autoreactive T cells in circulation often are scarce and the epitopes rely on the HLA genotype. In APS-1 patients, most of the effort has been carried out regarding 21-hydroxylase, where this autoantibody target has also shown to be targeted by autoreactive T cells [89], implying that the B and T cells collaborate on the autoimmune mechanisms causing pathology. In Aire ko mice, this has been shown for the interphotoreceptor retinoid-binding protein (IRBP) [90], as well as for BPI Fold Containing Family B Member 9 (BPIFB9) [79].

## 6. B Cells in AIRE-Deficient Mice Varies with Genetic Strain and Aire Mutations

AIRE-deficient mouse models have been indispensable in elucidating the molecular function of AIRE. Human AIRE and murine Aire share around 77% nucleotide homology in the coding regions and around 71% protein homology [91,92]. Several mouse strains like BALB/c, NOD (Non-Obese Diabetic) CD1, TRAMP (TRans-genic Adenocarcinoma of the Mouse Prostate), NIMR (Naval Medical Research Institute), and C57BL/6 mice have been used, and the phenotype of the disease varies from strain to strain (Reviewed in [93]). AIRE-deficient mice develop multiple features that resemble the human corresponding AIRE-deficient phenotype, like multiorgan lymphocytic infiltration, infertility, and the development of circulating autoantibodies against liver and sperm [94]. Nevertheless, even though some components of APS-1 are present, none of the main dyad of CMC, hypoparathyroidism, and Addison’s disease, or other apparent endocrinopathies are replicated, and the phenotype is in general surprisingly mild except on the NOD background [54]. This was among others shown by Hubert and colleagues [95], introducing 13-basepair deletion in Aire commonly seen in APS-1 patients on a C57BL/6 background, where they found an increase in activation of CD44highCD4+ T cells, while all other T cell subsets were unaffected. On the same genetic background but with Aire exon 2 and 3 deleted, T cells infiltrating the salivary gland, ovary, and eye, were found [20]. The immune reactions included sporadic infiltration into the sublingual salivary gland as well as severe infiltration in the parotid salivary glands, but no immune cells were seen in the stomach, liver, kidney, epididymis, thyroid, parathyroid, testis, ovary, and epidermis. Interestingly, the lymphocyte infiltration and disease were influenced by the age of these mice [20]. Some of the dominant-negative mutations causing APS-1 in humans have also been introduced in mice. Using mice on the NOD background, the mice showed spontaneous immune infiltrations in the liver, prostate, and salivary glands, with a partially inhibited TRA repertoire and developed neuropathy and early-onset diabetes [96,97], indicating the importance of quantitative changes in thymic antigen expression in the development of organ-specific autoimmunity.

Autoantibodies in AIRE-deficient mice have been detected in oocytes in the ovary, the outer layer of the retina in the eye, and in the parietal cells in the stomach [20]. Autoantibody reactivity is commonly targeting the liver and pancreas, testis, lung, eye, and salivary gland, but no autoantibodies against the major autoantigens in APS-1, including amongst others 21OH, 17OH, SSC, AADC, and TPH, were found in mice lacking Aire [20,98]. A few overlapping targets have however been found in both APS-1 patients and autoimmunity prone mice: The orthologue to NALP5 (MATER) is an autoantigen for a murine model of autoimmune ovarian failure [99,100,101], and anti-BPIFB1 (bactericidal/permeability-increasing fold-containing B1) are present in both APS-1 and one of the AIRE-deficient mouse models [79,102]. As the cytokine autoantibodies were discovered in human APS-1 patients in 2006 (against type I interferons) [80] and in 2010 (IL-17 and IL-22) [60,103], these autoantibodies were not analyzed in the first Aire ko mouse models. The first shared cytokine target between AIRE-deficient humans and AIRE-deficient mice was reported by Kärner et al., describing IgG1 neutralizing antibodies to IL-17A in aged AIRE-deficient BALB/c mice [104].

Although the Aire mouse models differ from humans in several aspects, they have provided invaluable insight into the establishment of immunological tolerance and holds potential for exploring new treatment options targeting the immune system.

## 7. Lessons from Other Autoimmune Diseases

Looking into autoimmune diseases in general, B cells have been found to play several diverse roles. These include both antibody-mediated as well as antibody independent mechanisms, it comprises their function as APCs, their secretion of inflammatory cytokines (e.g., TNF-α and IFN-γ), the modulation of antigen processing and presentation, and the generation of ectopic germinal centers (GCs) and tertiary lymphoid tissues at inflamed sites [105].

In some autoimmune disorders, autoantibodies have been shown to be directly pathogenic. One such example is Graves’ disease. In this disorder, the hyperthyroid state is triggered by autoantibody binding to and stimulating the thyrotropin (TSH) receptor, subsequently causing an increased release of thyroid hormones [106,107]. Autoantibodies can also exert an inhibitory effect on receptor functions as seen in Myasthenia gravis where they bind to acetylcholine receptors (AChRs) and efficiently block neurotransmission [108].

The importance of B cells as coordinators of the T cell functions is evident in several mouse models of autoimmune diseases. In a model of autoimmune hepatitis, known as a prototypic T cell-mediated disease, B cell depletion correlated with a decrease in the amount of CD4+ and CD8+ T effector cells with a significantly inhibited memory CD8+ T cell subset [109]. In mouse models of both rheumatoid arthritis and T1D, the lack of antigen-presenting B cells led to a milder disease highlighting the role of B cells in T cell activation [109,110,111]. B cells can also modulate antigen processing and presentation, thus indirectly affecting the subset of autoreactive T cells [112,113,114]. The secretion of inflammatory cytokines by B cells exerts a regulatory influence on T cells providing feedback stimulation leading to further B cell activation. This subsequently led to modulation of DC migration and activation of macrophages [115,116]. B cells can further be involved in lymphoid tissue genesis and the generation of ectopic GCs. This has been described for example in T1D, Graves’ disease, Sjögren’s syndrome and rheumatoid arthritis [117,118].

Recently, the identification of a B cell subpopulation with immunosuppressive capacity that downregulates immune responses and supports immunological tolerance has been characterized in several autoimmune diseases. Unlike Tregs, a specific transcription factor for regulatory B cells (Bregs) has not been discovered, and their identification relies solely on their capacity to inhibit T cell activation and cytokine secretion [119,120]. However, like for Tregs, the production of IL-10 and IL-35 are thought to be the main method of suppression [121]. In patients with T1D the IL-10 positive B cells are shown to produce less IL-10 in vitro [122], and IL-10 production from activated B cells delayed disease onset in young NOD mice [123]. Additionally, in autoimmune hyper- and hypothyroidism, Bregs are found to be decreased in numbers when compared to healthy controls [124], although functional studies are still needed to understand their detailed impact on pathology and tolerance. Importantly, Bregs can be induced by both adaptive and innate immune signals [125], and their functional ability to prevent autoimmune inflammation has been shown in mouse models [126]. Building on information from other autoimmune diseases might be a good strategy to understand APS-1 and vice versa.

## 8. Treatment Approaches Targeting B Cells in Autoimmune Diseases

Many current treatment options of autoimmune diseases that focus on B cells include monoclonal antibodies against surface markers which lead to B cell depletion. The most prominent examples are antibodies targeting CD19 (e.g., Obexelimab or Inebilizumab), CD20 (e.g., Rituximab) and CD22 (e.g., Epratuzumab). There are also further promising, but less used surface targets like CD52 (Alemtuzumab), which is efficient for the treatment of multiple sclerosis (MS) [127,128]. CD19 is expressed on B cells of all maturation stages from the pro-B cell stage until late stages of plasma cells, and classical monoclonal CD19 antibodies are for example applied for the treatment of MS (Inebilizumab) [129,130]. Targeting of CD20 leads to a depletion of mature naïve and memory B cells as wells as an inhibition of the development of short-lived plasma cells, although does not have as broad an effect as anti-CD19 treatment. Rituximab is used (partially off-label) for several autoimmune diseases like rheumatoid arthritis, T1D, MS, and systemic lupus erythematosus [131,132,133,134], but is also frequently used for research on B cells in autoimmune diseases. It was found to reduce IgM levels whilst not affecting levels of circulating IgG [135,136]. However, this does not apply to all autoimmune diseases since the reduction in IgG autoantibody titres has also been reported [137]. The antibody-independent effect of Rituximab is thought to be related to the elimination of B cells as APCs leading to a reduced stimulation of T cells [138,139].

Besides classical monoclonal antibody B cell depletion, other approaches can achieve an antigen-specific B cell depletion by redirecting T cells. This approach has been explored in Myasthenia gravis. This is a rare, but often severe, disorder of neuromuscular transmission causing fatigable muscle weakness. It is mostly due to antibodies directed against the AChR, but in a proportion of patients without AChR antibodies, antibodies to muscle-specific tyrosine kinase (MuSK) are found [140]. Like AChR, MuSK is also a membrane protein playing an essential role at the neuromuscular junction. By generating chimeric autoantibody receptor (CAAR) T cells expressing MuSK, MuSK reactive memory B cells were specifically eliminated by the CAAR T cells [141,142]. Other approaches aiming at a selective depletion of B cells are being developed, including the elimination of autoantigen-specific B cells by coupling autoantigens to magnetic nanoparticles and subsequent removal of these autoantigen-specific B cells through extracorporeal filtration [143]. The ultimate goal would be the selective depletion of B cells except for Bregs. Although still in its infancy, CD19-targeted CAR regulatory T cells have been established to suppress B cell pathologies. When injecting these into immunodeficient mice reconstituted with human PBMCs, recovery of graft-versus-host disease was observed [144].

Other treatment approaches focus on neutralizing factors involved in survival or activation of B cells, like BAFF, a proliferation-inducing ligand (APRIL), or transmembrane activator and calcium-modulator and cyclophilin ligand interactor (TACI) [145,146,147]. For example, elevated levels of BAFF have been observed in Sjogren’s syndrome, rheumatoid arthritis, and systemic lupus erythematosus. Inhibition of BAFF reduced the symptoms in autoimmune animal models [148,149], as well as in patients [145,146]. Belimumab, a recombinant human monoclonal antibody inhibiting BAFF, has amongst others been evaluated in an international trial involving 448 patients with systemic lupus erythematosus. Belimumab together with standard therapies for lupus nephritis enhanced renal responses and reduced the risk of renal-related events by 50% among patients [150]. There are currently 24 studies with Belimumab registered as “recruiting” or “not yet recruiting” at cliniclatrials.gov, most of which investigate systemic lupus erythematosus. Atacicept is a recombinant fusion protein of the binding portion of the TACI (transmembrane activator and CAML interactor) receptor that neutralizes BAFF and APRIL simultaneously. It is being tested in several autoimmune diseases, including MS and rheumatoid arthritis, but without any noticeable effects so far [151]. Inhibition of B cell activation by blocking the CD40/CD40L interaction of B and T cells was recognized as a possible target in a mouse model of lupus [152,153] but has not yet evolved into human trials.

**Figure 1:**
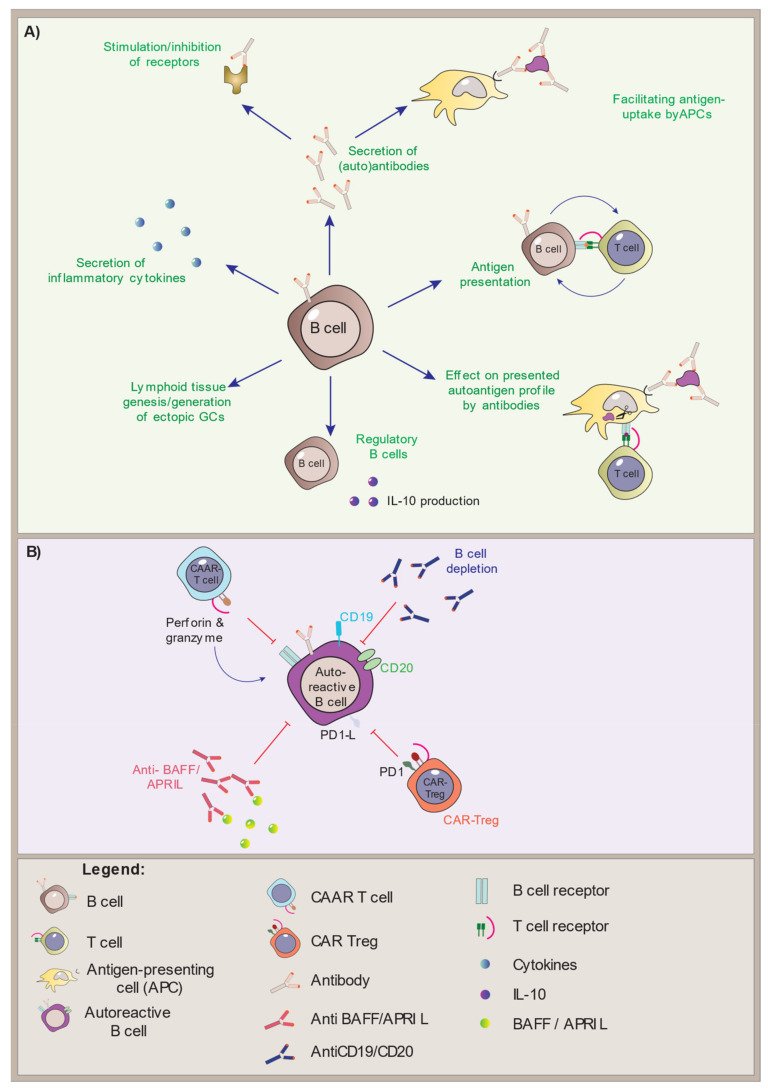
B cell function and modes of intervention. (**A**) The effector functions of B cells in the immune system, including the IL-10 producing regulatory B cells. (**B**) Modes of B cell depletion. Antibodies directed against the B cell-specific markers CD19 and CD20 will deplete all B cells, while chimeric auto-antibody receptor T (CAAR-T) cells and chimeric antibody receptor (CAR)-Tregs can be specifically targeted to autoreactive B cells.

Therapies have also been developed to induce apoptosis of B cell anergy by targeting the BCR or BCR-associated transmembrane signaling proteins like CD79 [154] or target kinases involved in BCR signaling [155], so far only tested in mouse model systems. Potential further targets might include inflammatory cytokines released by the B cells, like IL-6, TNF-α or IFN-α [145], or the lymphotoxin-β receptor to inhibit the formation of ectopic GCs [156] (Figure 1). The use and development of B cell-specific therapies will be interesting to follow and holds the potential to ameliorate several autoimmune disorders, providing treatment beyond the regular substitution medication.

## 9. Conclusions and Future Perspectives

APS-1 is a severe disease where mutations in the AIRE gene lead to the accumulation of autoimmune manifestations, mainly in the endocrine organs. It is a rare disease but has proved to be a powerful model shedding light on the mechanisms of thymic negative selection of T cells. Although the disease is mainly shown to be T cell-driven, accumulating evidence points to an aberrant immune reaction involving B cells. In particular, peripheral B cell tolerance is compromised resulting in the production of autoantibodies, aberrant T cell activation, and skewing of B cell populations. Modulation of B cell mechanisms has had beneficial effects on autoimmune diseases both in humans and mouse models, highlighting their potential for immunotherapies. They might have a better safety profile than T cell-directed therapies. Combining B and T cell-directed therapies is also a possibility. Nevertheless, detailed information on how the B cell subsets and BCR repertoire behaves in APS-1, functional studies, and explorations on how these cells react to different kinds of immune-modulatory drugs is crucial if we want to target the autoimmune process in APS-1 specifically, for example, to eliminate the self-reactive cells.

Research questions to prioritize:

- What is the inflammatory cytokine secretion profile of B cells in APS-1 patients?
- How do B cells in APS-1 patients interact with T cells, DCs, and macrophages, especially with regards to antigen presentation?
- Is the Breg subset functional in APS-1?
- Are the hallmark autoantibodies in plasma and sera from APS-1 patients pathogenic?
- Does B cell depletion therapy improve the main manifestations, and does it impact the interferon profile?
